# Contribution of Poland to Atmospheric Nitrogen Deposition to the Baltic Sea

**DOI:** 10.1007/s11270-018-4009-5

**Published:** 2018-10-25

**Authors:** Jerzy Bartnicki, Valiyaveetil Shamsudheen Semeena, Andrzej Mazur, Jerzy Zwoździak

**Affiliations:** 1 Norwegian Meteorological Institute, P.O. Box 43 Blindern, NO-0313 Oslo, Norway; 2 Institute of Meteorology and Water Management—National Research Institute, ul. Podleśna 61, PL-01-673 Warsaw, Poland; 3 General Tadeusz Kosciuszko Military University of Land Forces MULF, ul. Czajkowskiego 109, 51-147 Wrocław, Poland

**Keywords:** Nitrogen emissions, Atmospheric transport, Nitrogen deposition, Baltic Sea, Air pollution model

## Abstract

Poland is the second most important emission source after Germany in contributing atmospheric nitrogen deposition to the Baltic Sea basin. The main sectors contributing to reactive nitrogen emissions from Polish sources, in the period 1995–2014, are combustion and transportation, responsible together for over 97% of nitrogen oxide emissions, and agriculture responsible for over 98% of ammonia emissions. The EMEP MSC-W model with 50-km resolution was used for estimating the contribution of nitrogen emission sources from Poland to nitrogen deposition into the Baltic Sea basin and its sub-basins, in the period 1995–2014. Polish contribution in this period is mainly visible in annual wet deposition of reduced nitrogen with the range 13–18% and in wet deposition of oxidized nitrogen: 9–15%. Concerning sub-basins, a major contribution for Polish sources to total nitrogen deposition can be noticed for Baltic Proper with the range 13–19%, followed by northern sub-basins (7–18%) and finally by three western sub-basins (5–7%). Polish contribution to the Baltic Sea Basin in the year 2013 was analyzed in more detail using two models, the EMEP MSC-W model with 50-km resolution and model developed at the Institute of Meteorology and Water Management in Warsaw with 14-km resolution (IMWM Model). Both models give similar results concerning the deposition of oxidized nitrogen from Polish sources, but results show that the deposition of reduced nitrogen calculated with IMWM model is lower. The most likely reasons for the differences are different parameterizations of the deposition processes and chemical reactions in both models.

## Introduction

Eutrophication is one of the most serious environmental problems for the Baltic Sea at present (Andersen et al. 2009). It is mainly caused by input of nutrients to the Baltic Sea basin with reactive nitrogen and phosphorus playing a major role in this process. The reduction of nutrients input to the sea has been an important goal of the Convention for the Protection of the Marine Environment of the Baltic Sea Area (HELCOM) since its establishment in 1974 (Heidam 1993; HELCOM 1987, 1989, 1993, 1997). HELCOM has been compiling available information about the sources of the amount of nutrient inputs into the Baltic Sea since the mid-1980s. In this way, HELCOM has been able to follow the progress toward reaching politically agreed nutrient reduction input goals.

In case of phosphorus, waterborne input accounts for more than 90% of the total input to the Baltic Sea (Svendsen et al. 2015) and its atmospheric input is relatively low (Krom et al. 2004). However, later estimates indicate that atmospheric phosphate deposition can sustain up to 38% of new production during summer and autumn in the eastern Mediterranean (Markaki et al. 2003).

For a long time, it has been assumed that the waterborne input, mostly riverine input, is the most important contributor to deposition of nitrogen into the Baltic Sea, and the airborne part can be neglected. The recent results (Bartnicki 2014; Svendsen et al. 2015) indicate that the contribution of airborne to total (airborne + waterborne) nitrogen input into the Baltic Sea basin is also important and relatively high, accounting for 21–31% for the period 1995–2010 (Ruoho-Airola et al. 2012). In this comparison, the airborne contribution was calculated from the results of the EMEP MSC-W model (Bartnicki 2014) and the information about waterborne input into the Baltic Sea taken from Wulff et al. (2009). Since then, atmospheric input of nitrogen accounts for approximately one quarter of total nitrogen input and it is routinely monitored by HELCOM based on measurements and model results.

Meteorological Synthesizing Centre–West (MSC-W), as a part of the European Monitoring and Evaluation Programme (EMEP), has been working closely with HELCOM on modeling nitrogen deposition to the Baltic Sea since 1997. EMEP is a scientifically based and policy-driven international program under the Convention on Long-range Transboundary Air Pollution (CLRTAP) with the aim to solve transboundary air pollution problems. In the frame of the joint HELCOM-EMEP long-term project, annual deposition of nitrogen to the Baltic Sea basin and its sub-basins are calculated every year using the latest available emission inventories for all EMEP sources (Bartnicki et al. 2017). Not only nitrogen depositions are calculated in the project but also depositions of selected heavy metals and persistent organic pollutants as well, which is a task of the Meteorological Synthesizing Centre East of EMEP (MSC-E). In addition, concentrations and depositions calculated every year by MSC-W and MSC-E are compared with the measurements available at HELCOM stations and compiled by the Chemical Coordinating Centre (CCC) of EMEP. In addition to nitrogen calculations, the role of MSC-W in the EMEP-HELCOM project is a coordination of the three EMEP Centers working and reporting to HELCOM.

Atmospheric deposition of nitrogen to the Baltic Sea basin was estimated in several studies (Heidam 1993; Hertel et al. 2003; Bartnicki and Fagerli 2008; Langner et al. 2009; Bartnicki et al. 2011; Hongisto 2011; Geels et al. 2012; Bartnicki 2014). In all these studies, wet deposition of nitrogen dominates dry deposition, and oxidized nitrogen deposition is higher than that of reduced nitrogen. All these studies also show that in the modeled deposition of both oxidized and reduced nitrogen, a strong south–north gradient across the Baltic Sea region is present, declining by up to an order of magnitude from Denmark to the northern part of Sweden (Langner et al. 2009).

In several studies, the contributions of different countries to nitrogen deposition in the Baltic Sea basin have been assessed. Geels et al. (2012) estimated, using the DEHM model, that the nine HELCOM countries bordering the Baltic Sea contribute approximately 50% of the nitrogen deposition in 2007, with Germany being the largest single contributor. Bartnicki et al. (2011), using the EMEP MSC-W model, found even greater contribution of HELCOM countries, with Germany to be the single largest contributor followed by Poland.

Results of the recent EMEP project for HELCOM, in which source allocation budget for nitrogen was calculated (Bartnicki et al. 2016), confirm that Germany is the number one source of atmospheric nitrogen input to the Baltic Sea basin followed by Poland as the number two contributor for all years in the period 1995–2014. However, for some of the sub-basins of the Baltic Sea and especially for the largest sub-basin, Baltic Proper, Poland is the major contributor to nitrogen deposition. It should also be mentioned that Poland is the lead source in waterborne input into the Baltic Sea (Svendsen et al. 2015).

In this study, we have focused on different aspects of Polish contribution to atmospheric nitrogen deposition to the Baltic Sea. The period 1995–2014 was selected for the analysis, although the EMEP model was also run for the year 2015. However, a different model grid (0.1 × 0.1 deg) was used for 2015 (Fagerli et al. 2017), which is not consistent with the grid used for the years 1995–2014 (50 km × 50 km). For one specific year, 2013, we have also compared results of the EMEP model with the results of another model developed at the Institute of Meteorology and Water Management (IMWM) in Poland (Mazur 2008, 2016; Mazur et al. 2014).

## Methods

This study consists of two parts. In the first part, annual nitrogen depositions to the Baltic Sea basin and its nine sub-basins were calculated for the period 1995–2014. These calculations have considered all EMEP emission sources, including Polish sources. Polish contribution was calculated as absolute (mass of nitrogen deposited) and relative, in percent of total deposition from all EMEP sources.

In the second part of the study, we have focused on one year currently available in the EMEP data base—2013. For the same year in addition to standard EMEP MSC-W model with 50-km resolution, we have also used the air pollution model developed at the Institute of Meteorology and Water Management (IMWM) in Poland (Mazur 2008; Mazur et al. 2014) with better spatial resolution—14 km. The year 2013 was chosen because this was the year for which the results of IMWM model were available.

### The EMEP/MSC-W Model

The EMEP/MSC-W model is a multi-pollutant three-dimensional Eulerian Chemical Transport Model, which has been used for nitrogen computations presented here for the period 1995–2014. The model considers the most important processes of emissions, advection, turbulent diffusion, chemical transformations, wet and dry depositions, and inflow/outflow of pollutants into/out of the model domain. The EMEP/MSC-W model has been documented in detail in Simpson et al. (2012) and in the annual chapters on model updates in the EMEP status reports (Tsyro et al. 2014; Simpson et al. 2015, 2016, 2017; Fagerli et al. 2017).

The model is regularly evaluated against measurements from the EMEP network under the LRTAP convention (e.g., Gauss et al. 2017a, 2017b; Tsyro et al. 2017). In addition, it has also been evaluated in many international research projects and operational services (e.g., Copernicus Atmosphere Monitoring Service, http://macc-raq-op.meteo.fr/). The performance of the EMEP/MSC-W model can be considered as state-of-the-art over a large range of both gaseous species and particulate matter. The model code (software) is also available as Open Source (https://github.com/metno/emep-ctm) and has been widely used both as a research tool and for underpinning of air quality legislation.

The EMEP/MSC-W model version rv4.15 has been used for the deposition calculations (Fagerli et al. 2017). This version can be run on many different spatial resolutions, including 0.1 × 0.1 degree and 50 × 50 km. Here, the model version with 50 km × 50 km resolution was used for all model runs since there are only few years with available meteorological data for the high-resolution version.

**Fig. 1:**
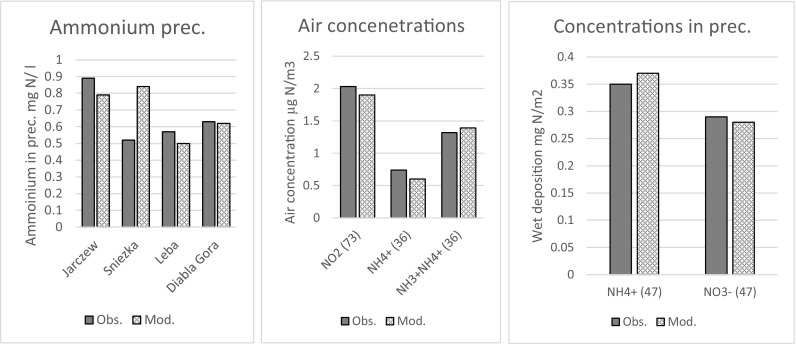
Comparison of EMEP model results with measurements for the year 2013 averaged over all EMEP sites with available measurements, including Polish stations. In brackets number of stations used for calculations. Concentration of ammonium in precipitations is also compared at four individual Polish stations

Comparison of EMEP model results with all measurements available at EMEP stations for the year 2013 are shown in Fig. 1. These stations include both HELCOM sites used for evaluation of nitrogen deposition to the Baltic Sea. In addition, comparison of annual 2013 ammonium concentration in precipitation is shown for four Polish stations in Fig. 1.

The agreement between EMEP model results and measurements averaged over all stations is very good both for air concentrations and concentrations in precipitation. This agreement is also good for concentrations of ammonium in precipitations measured in three out of four Polish stations. Only for Sniezka station the model overestimates measurements by approximately 60%. The main reason for this overestimation is an elevated (ca. 1600 m) location of this station and not good enough resolution of vertical distribution of computed concentrations.

### The IMWM Model

The IMWM model is a multi-pollutant three-dimensional Eulerian Chemical Transport Model. In this study, it has been used for nitrogen computations for one year, 2013, only. This model is developed at the Institute of Meteorology and Water Management in Warsaw, Poland (Mazur 2008; Mazur et al. 2014). The IMWM model takes into account the same physical and chemical processes as the EMEP/MSC-W model, but the parameterizations of these processes are slightly different. This multipollutant model considers not only acidifying agents but also radioactive pollutants and heavy metals as well. However, here we are only interested in application of the IMWM model for simulation of atmospheric transport and deposition of nitrogen.

The computational domain of the IMWM model is flexible and can be defined by the user depending on available meteorological input. For the present application, the model domain was the same as the domain of the Numerical Weather Prediction (NWP) model COSMO (Consortium for Small-Scale Modeling), which is currently operational at IMWM. The grid size of the COSMO domain is 193 × 161 nodes and it covers most of Europe including the entire territory of Poland and Baltic Sea region. The spatial resolution of this domain is 14 km. The terrain following vertical coordinate is used in the model with flexible definition of vertical levels. The typical number of vertical levels, also used in this application, is 21 with the domain top at 5 km.

Annual emissions of nitrogen oxides, ammonia, and sulfur dioxide are used as input to acidification part of the IMWM model. The parameterization of so-called local deposition is introduced in the IMWM model to account for much higher concentrations close to the point source compared to average concentration in the grid where the source is located. Also, the elevation of the sources is considered for correct vertical distribution of emissions.

Three-dimensional advection–diffusion equation describes sources, atmospheric transport, chemical transformations, and depositions of pollutants in the Eulerian IMWM model. The Fractional Steps method (Yanenko 1971) is used for solution of different parts of the basic equation. Area Flux Preserving method (Bott 1989a, b) is used for advection part of the equation and semi-implicit Crank–Nicholson method (Potter 1973) for the numerical solution of the diffusion part.

Chemistry of the IMWM model is similar to one of the earlier versions of the EMEP model (Barrett and Berge 1996). Chemical reactions include 10 components: NO, NO_2_, PAN, HNO_3_, NH_4_NO_3_, NH_3_, (NH_4_)_2_SO_4_, SO_4_, and SO_2_. In this chemical scheme, the chemical reactions of nitrogen and sulfur are interconnected only by the presence of ammonia. All reaction constants are taken from Barrett and Berge (1996).

Pollutants are removed from the air by dry and wet deposition. The resistance analogy method is used for parameterization of dry deposition and scavenging rates for parameterization of wet depositions. It is assumed that both dry and wet deposition occur at the same time and the total deposition is calculated first and then partition to dry and wet deposition is calculated. The dry deposition coefficients and scavenging ratios are taken from Barrett and Berge (1996).

**Fig. 2:**
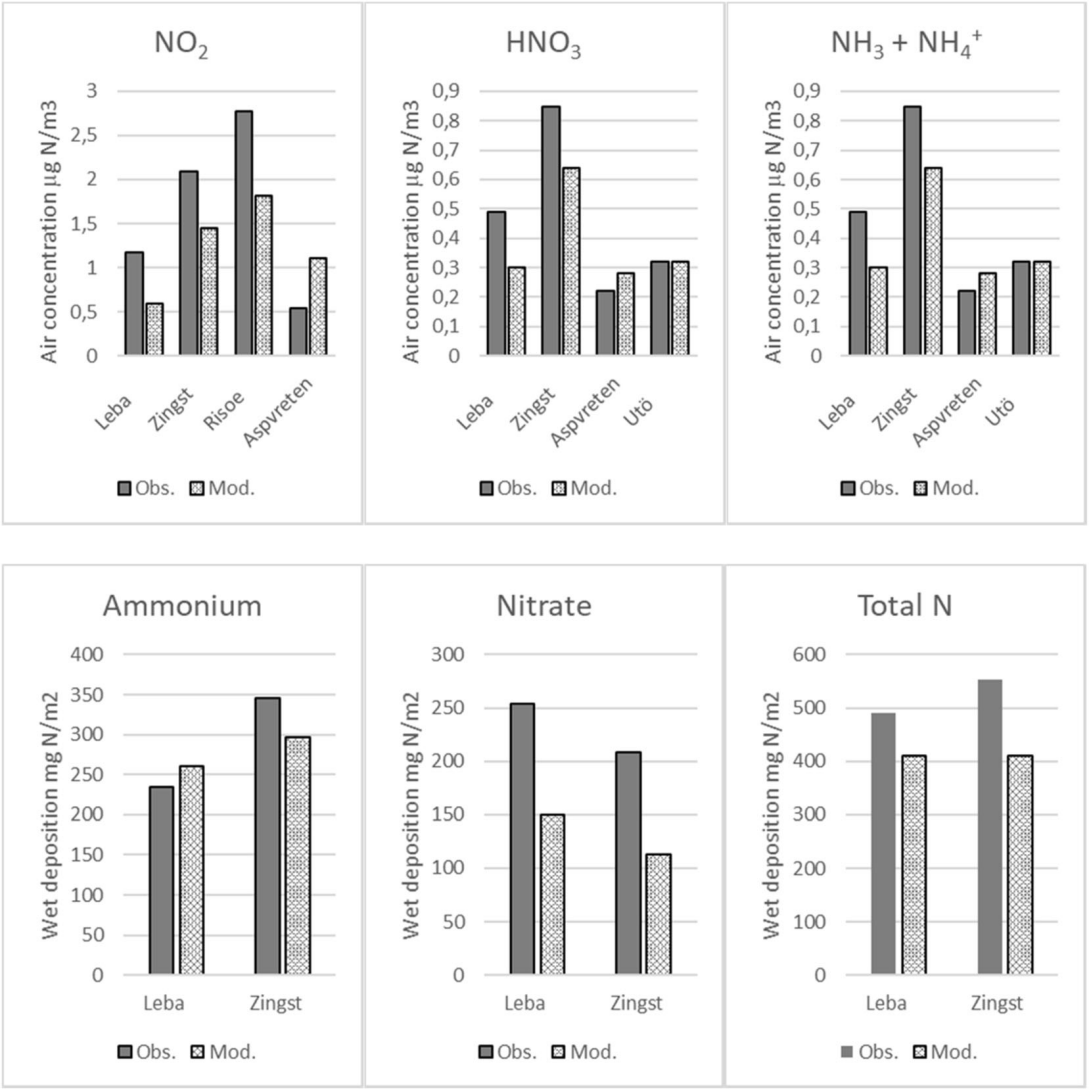
Comparison of IMWM model results with measurements for the year 2013 in selected sites belonging to HELCOM database

The results of IMWM model for acidifying compounds have been compared with available observations in the model domain with the focus on measurements from Polish sites. This comparison indicates that the IMWM slightly underestimates nitrogen oxide concentrations in the air with the typical correlation coefficient of the order of 0.6. However, the model results are within a factor of two with the measurements. For this study, the results of the IMWM model were compared with measurements for selected HELCOM stations used for evaluation of nitrogen deposition to the Baltic Sea in the year 2013. This comparison is shown in Fig. 2.

There is a relatively good agreement for deposition of ammonium, whereas some model underestimation can be noticed for nitric acid and total nitrogen. Air concentrations of NO_2_ measured at three stations, Leba, Zingst, and Risoe, are underestimated by the model, whereas these were overestimated at Aspvreten. However, measured and modeled concentrations of nitric acid and ammonia + ammonium are in good agreement in two stations: Aspvreten and Utö.

## Nitrogen Emissions from Poland

We start with a short review of major processes, or rather emission sectors, responsible for nitrogen oxides and ammonia emissions from Poland and other countries as well. Nitrogen emissions used as input for the EMEP MSC-W model are available from the EMEP Centre for Emission Inventories and Projections (CEIP) located in Vienna, Austria. For the modeling purpose, they are assigned to 11 emission sectors, called SNAP sectors (CEIP 2018), which are explained in Table 1.

**Table 1:** List of the 11 SNAP emission sectors as specified in the EMEP-CORINAIR Emission Inventory Guidebook

SNAP sector	Content
Sector 1	Combustion in energy and transformation industry
Sector 2	Non-industrial combustion plants
Sector 3	Combustion in manufacturing industry
Sector 4	Production processes
Sector 5	Extraction and distribution of fossil fuels and geothermal energy
Sector 6	Solvent and other product use
Sector 7	Road transport
Sector 8	Other mobile sources and machinery (including ship traffic)
Sector 9	Waste treatment and disposal
Sector 10	Agriculture
Sector 11	Other sources and sinks

Emissions of all components, including nitrogen, used as input to the EMEP MSC-W model are reported by all EMEP Contracting Parties every year and are available later on the web (CEIP 2018). Emissions of all components included in the EMEP MSC-W model, and especially sulfur components, can influence the calculated nitrogen depositions because of non-linear chemistry in the model equations (Simpson et al. 2012). However, the most important contributor for nitrogen depositions are the nitrogen emissions.

### Main Sources of Polish Nitrogen Emissions

In case of Polish nitrogen emissions, the most important sources of nitrogen oxides emissions are combustion and transportation. The SNAP classification with 11 emission sectors was used by the EMEP MSC-W model for the period 1995–2014. In this classification, combustion includes sector 1—combustion in energy and transformation industry, sector 2—non-industrial combustion plants, and sector 3—combustion in manufacturing industry. Transportation includes sector 7—road transport and sector 8—other mobile sources and machinery (including ship traffic). Polish ammonia emissions are dominated by only one important sector: sector 10—agriculture. The rest of the sectors are assigned to the category “other.”

**Fig. 3:**
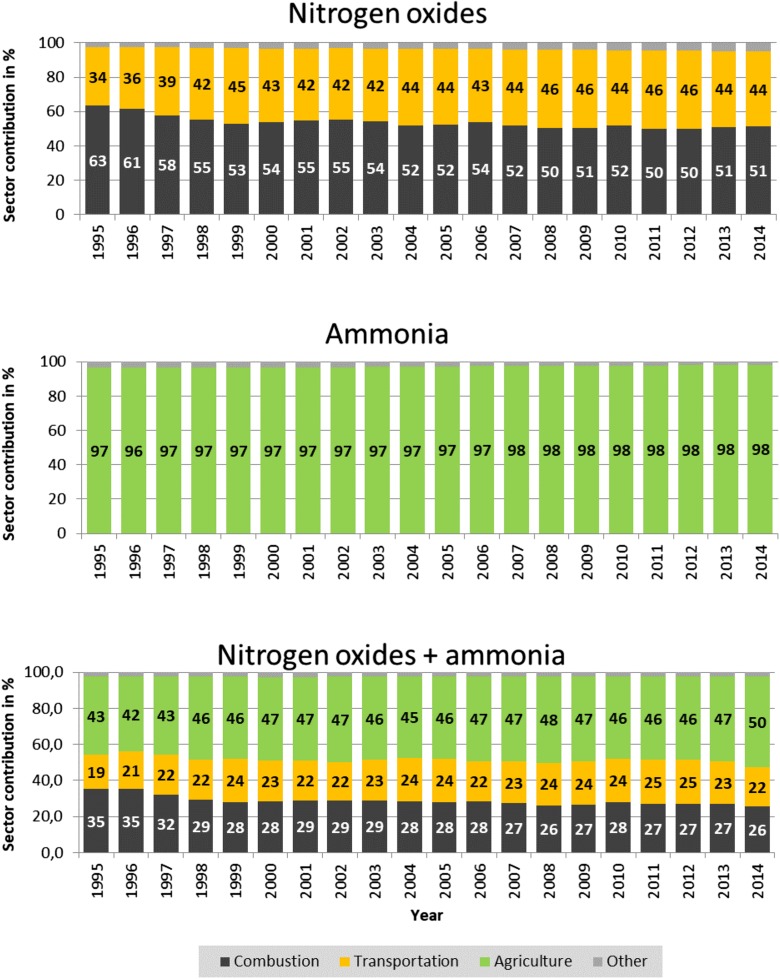
Contributions of different emission categories to annual nitrogen oxides, ammonia, and total nitrogen emissions from Polish sources in the period 1995–2014

Contributions of selected emission categories to annual nitrogen oxides, ammonia, and total nitrogen emissions from Polish sources in the period 1995–2014 are shown in Fig. 3.

Combustion is the main source of nitrogen oxide emissions from Poland, responsible for 51–54% of the total nitrogen emissions in the considered period. Concerning individual SNAP sectors, emissions from sector 1 are the major part (64–70%) of the combustion emissions, followed by sector 2 (15–23%) and sector 3 (11–17%).

Transportation is the second large source of emissions in Poland, which accounts for 42–46% of nitrogen oxide emissions. Combustion and transportation together are responsible for more than 95% of nitrogen oxide emissions from Poland. The contributions of all other sources are much lower, below 5% for all considered years.

Agriculture is dominating annual ammonia emissions from Polish sources in the period 1995–2014. Contribution of agriculture to annual ammonia emissions is quite stable, accounting for 97–98% of ammonia emissions from Poland. Contribution of all other emission sources of ammonia is very low, below 3%, for all considered years.

It is also interesting to examine the contributions of selected emission categories to total nitrogen emissions (nitrogen oxides + ammonia) from Polish sources. Figure 3 shows that the major category contributing to total nitrogen emissions in the period 1995–2014 is agriculture (45–48%). Combustion is the second on the list with 26–29% contribution. Also, transportation contributes significantly to total nitrogen emissions from Poland in the range 22–25%. Annual contributions of these categories are relatively stable over the entire period, not changing more than 4%.

### Nitrogen Emissions in the Period 1995–2014

**Fig. 4:**
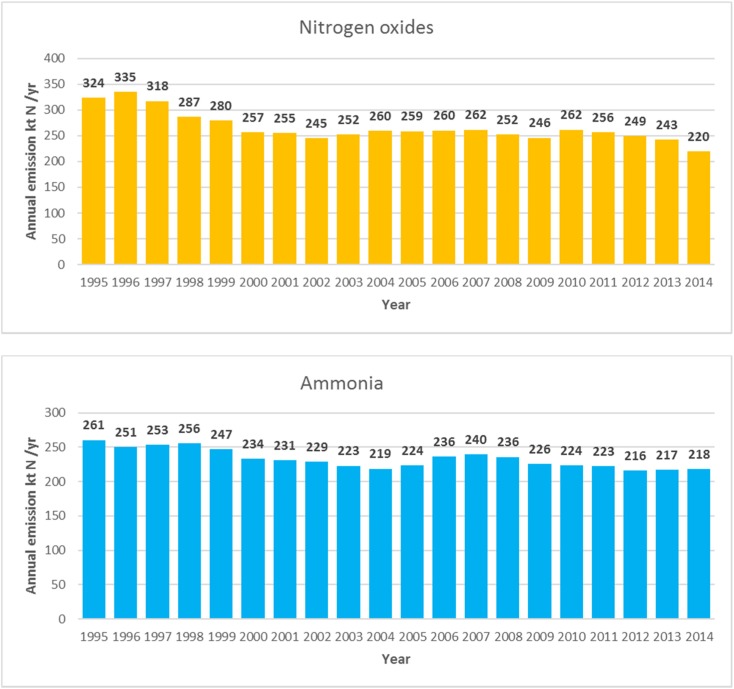
Annual emissions of nitrogen oxides and ammonia from Polish sources in the period 1995–2014

Annual emissions of nitrogen oxides and ammonia from Polish sources in the period 1995–2014 are shown in Fig. 4.

Both, annual nitrogen oxide emissions and annual ammonia emissions from Poland are changing slightly from one year to another, more for nitrogen oxides than for ammonia. Polish annual nitrogen oxide and ammonia emissions in 2014 are 32 and 16% respectively lower than the corresponding annual emissions in the year 1995. The maxima of annual emissions can be noticed in the years 1996 (335 Mg N) and 1995 (261 Mg N) for nitrogen oxides and ammonia, respectively. In the latest available year 2014, annual emissions of nitrogen oxides and ammonia are 220 Mg N and 218 Mg, respectively.

At the beginning of the period, in 1995, total nitrogen emissions from Poland accounted for 584 Mg N. Maximum of total nitrogen emissions from Poland occurred in 1997 (591 Mg N) and total nitrogen emissions decreased to 438 Mg N towards the end of the period, in 2014. The reduction of annual total nitrogen emissions between 1995 and 2014 is 25%.

A similar temporal pattern of nitrogen emissions like in Poland can be observed in the remaining HELCOM Contracting Parties in the considered period. Nitrogen oxide emissions are reduced in all HELCOM Contracting Parties in the range of 12% (Russia) to 61% (Denmark). Concerning ammonia, annual emissions increased in the considered period in three countries: Estonia (10%), Germany (9%), and Finland (2%). They declined in the remaining HELCOM countries in the range 2–33%.

### Distribution of Polish Nitrogen Emissions

**Fig. 5:**
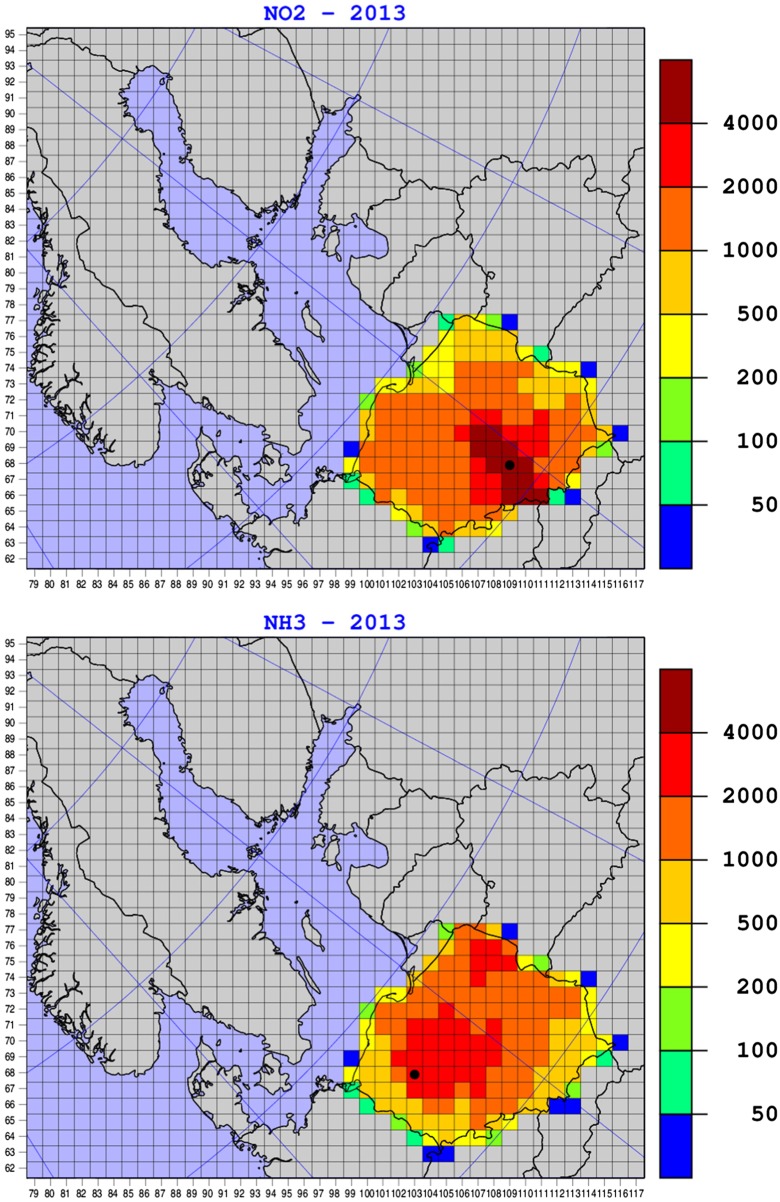
Maps of annual nitrogen oxides and ammonia emissions from Polish sources in 2013, in the EMEP grid system. Units: kt N per year and per grid. A grid with maximum location is marked with a black circle

Maps of annual nitrogen oxides and ammonia emissions from Polish sources in 2013 are shown in Fig. 5. These maps are presented in the numerical grid system of the EMEP MSC-W model with 50-km resolution. The grids with maximum locations are marked with a black circle. The spatial distribution of nitrogen oxide and ammonia emissions in Poland is very stable over the entire period 1995–2014 because of the same location of the major emission sources. We present the maps for the year 2013 because it was used for the comparison of two model results described in Chapter 5.

The maxima of Polish nitrogen oxides emissions are visible in central Poland, where the road traffic is the most intensive, and in the south of Poland (Silesia region), where, in addition to intensive traffic, the major combustion sources are located. Absolute maximum of annual nitrogen oxide emissions in 2013 is in the model grid (109, 68), which includes Katowice city. Except for the belt of several grids with elevated emissions, emissions from the rest of the Polish territory are relatively uniform, with slightly lower emission levels to the East.

Ammonia emissions are more uniformly distributed among Polish territory than nitrogen oxide emissions, following relatively uniform distribution of agricultural activities. Slightly elevated levels of ammonia emissions can be noticed in central Poland and close to the eastern border of Poland. Maximum of annual Polish ammonia emissions in 2013 is located in the model grid (103, 68) near the city of Poznan. Polish ammonia emissions are slightly decreasing towards the western and south eastern border of Poland. Maxima of ammonia emissions are lower than maxima of nitrogen oxide emissions from Polish sources. In addition, an important difference between nitrogen oxide and ammonia emissions is a strong seasonal variability of ammonia emissions and relatively more flat profile of nitrogen oxide emissions. Polish ammonia emissions are significantly higher in the growing and especially fertilizing season (March–April) than in the remaining months of the year. Temporal distribution of nitrogen oxide emissions is described by two factors: monthly and daily. Emissions from SNAP sector 1 are mainly dependent on the seasons of the year and show maximum in winter. SNAP sector 2 consists mainly of domestic combustion and shows large day-to-day variation depending on heating. Traffic emissions are relatively flat throughout the year. Seasonal changes in nitrogen oxide emissions differ between the individual EMEP countries. In case of Poland, the difference between winter and summer emissions of nitrogen oxides is of the order of 10%.

### Nitrogen Emission Inventories in the IMWM Model

For comparison of the results of EMEP MSC-W and IMWM models, with different resolutions, we kept the annual nitrogen emissions in 2013 the same for both models. This rule was applied not only to nitrogen emissions but also to emissions of all other components necessary to run the IMWM model. The emission inventories for IMWM model were interpolated from the EMEP grid with 50-km resolution to the IMWM grid with 14-km resolution, preserving the mass. Therefore, the spatial distribution of nitrogen emissions used by the IMWM model is the same as spatial distribution of nitrogen emissions used by the EMEP MSC-W model shown in Fig. 5.

## Contributions of Poland to Nitrogen Deposition in the Period 1995–2014

In the period 1995–2014, Poland is the number two contributor to atmospheric deposition of all kinds (oxidized, reduced, total) to the Baltic Sea (Bartnicki et al. 2016). In the frame of a long-term cooperation between EMEP and HELCOM, not only nitrogen depositions to the Baltic Sea basin are calculated each year but also depositions to sub-basins of the Baltic Sea.

### Nitrogen Deposition to the Entire Baltic Sea Basin

**Fig. 6:**
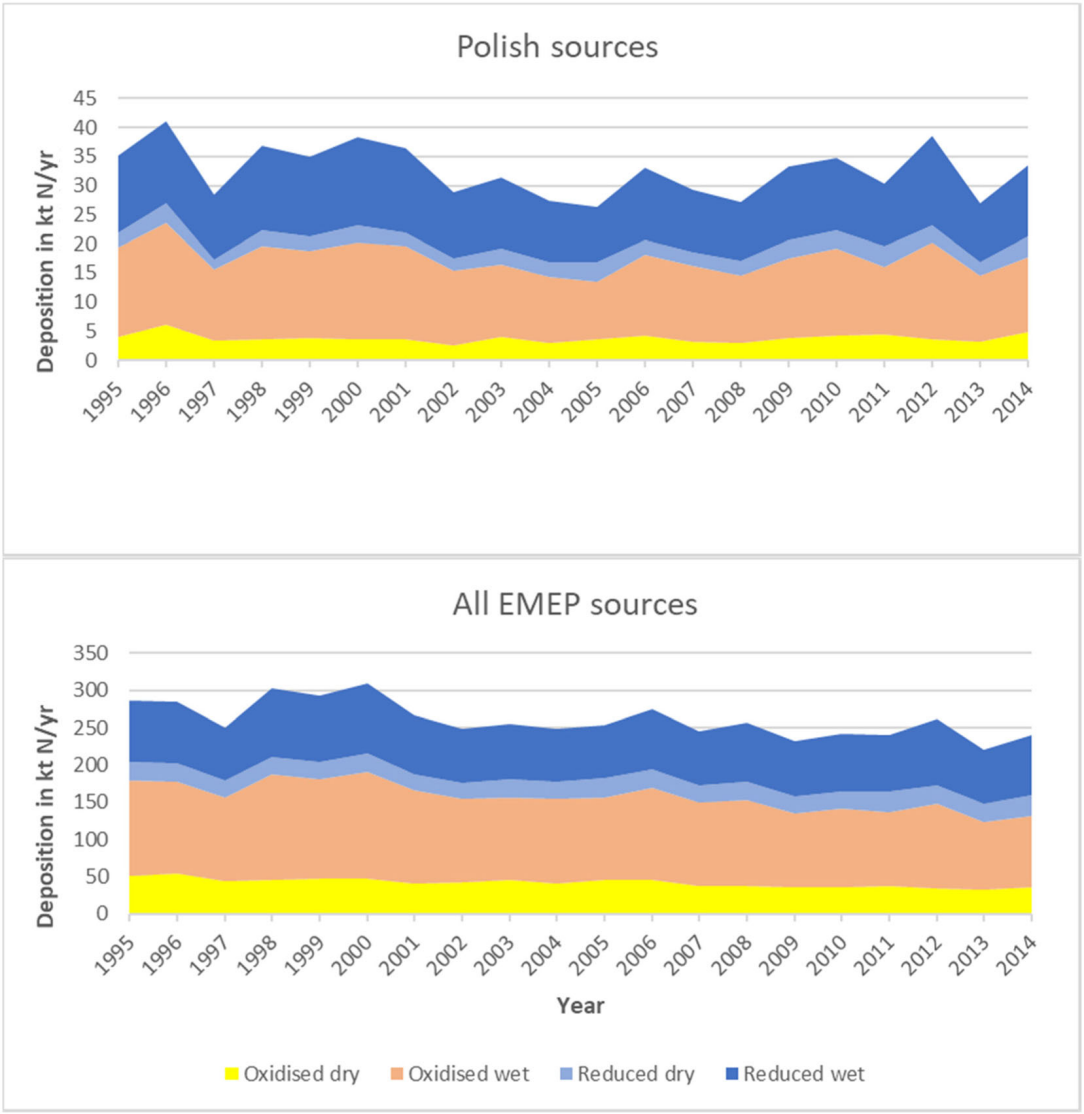
Annual dry and wet depositions of oxidized and reduced nitrogen to the Baltic Sea basin. Depositions from all EMEP emission sources (below) and depositions from only Polish emission sources (above) for the period 1995–2014

Annual dry and wet depositions of oxidized and reduced nitrogen to the entire Baltic Sea basin are shown in Fig. 6 for the period 1995–2014. Depositions from all EMEP emission sources are shown in Fig. 6, together with depositions resulting from only Polish emission sources.

Wet deposition dominates all kinds of nitrogen depositions to the Baltic Sea basin from all EMEP emission sources. On average, over the considered period, annual wet deposition accounts for 74% of annual total nitrogen deposition, with the range 72–77% depending on the year. Wet deposition of oxidized nitrogen is the main contributor to total nitrogen deposition for each year of the considered period with the range 40–47%, followed by wet deposition of reduced nitrogen with the range 28–34%.

Maxima of annual deposition of oxidized, reduced, and total nitrogen occur in the same year 2000. Minima of oxidized and total deposition are visible at the end of the period in the year 2013, whereas maximum of reduced deposition takes place in the year 2004.

All kinds of annual nitrogen depositions from all EMEP emission sources are lower in 2014 than in 1995. A relatively large decline can be noticed for deposition of oxidized nitrogen, 30% for dry deposition and 26% for wet deposition. In case of reduced nitrogen, there is only 3% decline in dry deposition, whereas there is a 16% increase in wet deposition. Annual deposition of total nitrogen to the Baltic Sea basin declines from 287 kt N in the year 1995 to 240 kt N in 2014, which corresponds to 17% reduction.

Concerning contributions of different kinds of depositions to total nitrogen deposition into the Baltic Sea basin, the proportions are quite similar when only contributions from Polish emission sources are considered. Wet deposition dominates again and even more in this case with 80% average contribution over the considered period with the range 73–83%. Wet deposition of oxidized nitrogen is the main contributor to total nitrogen deposition for each year of the considered period with the range 37–45%, followed by wet deposition of reduced nitrogen (34–40%), dry deposition of oxidized nitrogen (9–15%), and dry deposition of reduced nitrogen (6–13%). Maxima of annual depositions are mostly visible at the beginning of the considered period. For oxidized nitrogen, maxima of dry deposition (6.2 kt N) and wet deposition (17.4 kt N) occur in 1996. Maximum dry deposition of reduced nitrogen (3.5 kt N) and maximum of total nitrogen deposition (41.1 kt N) take place in the same year 1996. Only wet deposition of reduced nitrogen has a maximum (15.2 kt N) in another year—2012. Minima of wet deposition of both oxidized and reduced nitrogen can be seen in the same year 2005, with 9.9 kt N for oxidized nitrogen and 9.5 kt N for reduced nitrogen. Minima of dry deposition can be noticed in the year 2002 (2.7 kt N) for oxidized nitrogen and in the year 1997 (1.8 kt N) for reduced nitrogen.

Compared to the year 1995, there is a decline in wet deposition of nitrogen from the Polish sources in the year 2014: 15% for oxidized nitrogen and 9% for reduced nitrogen. On the other hand, there is an increase in dry deposition of oxidized nitrogen (17%) and a significant increase of 42% in dry deposition of reduced nitrogen. Deposition of annual total nitrogen to the Baltic Sea basin from Polish sources is also decreasing from 35.2 kt N in 1995 to 33.5 kt N in 2014, which accounts for 5% reduction.

**Fig. 7:**
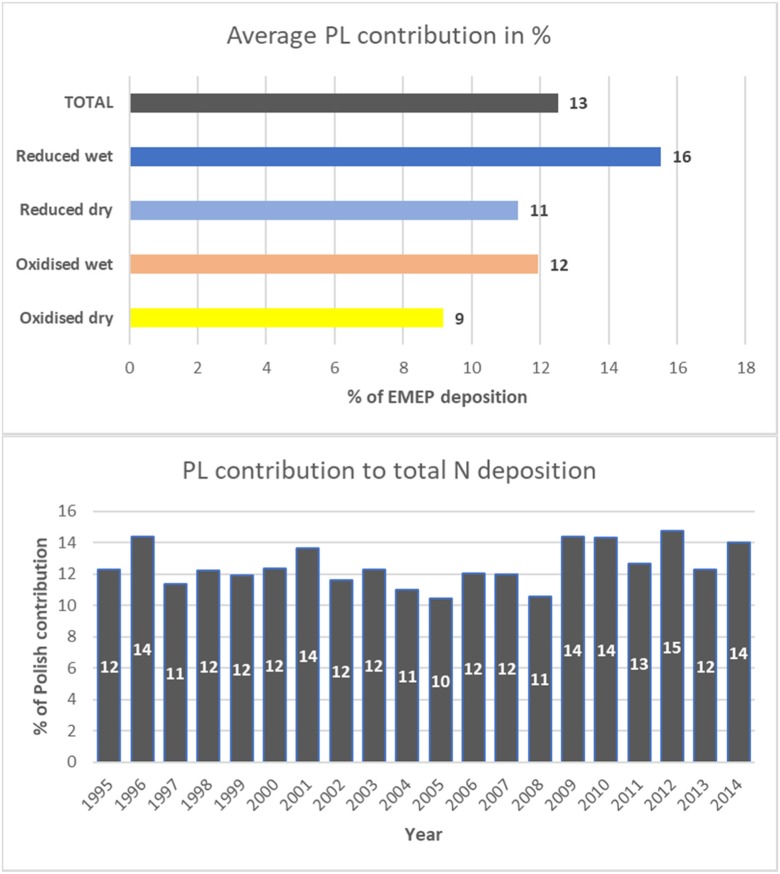
Average (1995–2014) contribution of Polish sources to different kinds of annual nitrogen deposition to the Baltic Sea basin (above). Annual contributions of Polish sources to total nitrogen deposition to the Baltic Sea basin in the period 1995–2014 (below). Both contributions in per cent of nitrogen deposition from all EMEP sources

Relative contributions of Polish sources to annual nitrogen depositions to the Baltic Sea basins are presented in Fig. 7 as average values for the period 1995–2014. Depositions from Polish sources are expressed in percent of total nitrogen depositions from all EMEP emission sources. In addition, relative annual contributions of Polish emissions to total nitrogen deposition to the Baltic Sea basin are also shown in Fig. 7.

The main contribution of Polish sources, in the period 2000–2014, is visible in wet deposition of reduced nitrogen with the range 13–18% and average contribution 16%. The lowest contribution from Polish sources can be seen in dry deposition of oxidized nitrogen with the range 6–13% and average value 9%. Contribution from Polish sources to annual deposition of total nitrogen is within the range of 10–15%, with minimum in 2005 and maximum in 2012. On average, over the entire period, Polish contribution to total nitrogen deposition is 13%, with slightly higher values at the end of the period starting in 2009. In general, Polish contribution of reduced nitrogen deposition into the Baltic Sea is higher than that of oxidized nitrogen deposition with the wet deposition being higher than the dry deposition.

### Nitrogen Depositions to Sub-Basins of the Baltic Sea

**Fig. 8:**
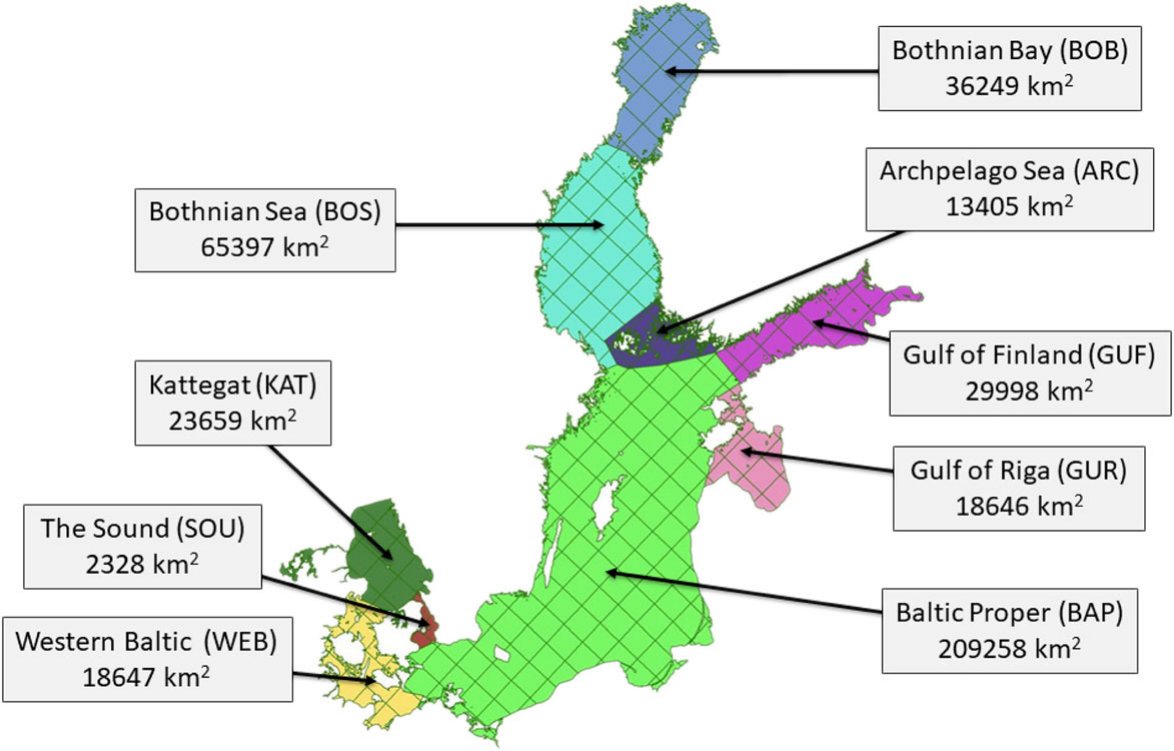
Locations of sub-basins of the Baltic Sea basin. In addition to names, acronyms and surface areas of sub-basins are also shown

Deposition of nitrogen to the sub-basins of Baltic Sea is discussed in detail in this section. The nine sub-basins which are important for HELCOM are shown in Fig. 8, together with the grid system of the EMEP MSC-W model with 50-km resolution.

There are large differences in the size of different sub-basins, with the largest one being Baltic Proper, covering an area of 209,258 km^2^ and 126 EMEP grid squares. The area of the smallest sub-basin—The Sound—is almost two orders of magnitude smaller (2328 km^2^). The Sound sub-basin covers only eight EMEP grid squares. The size of individual sub-basin has a significant influence on the annual input of nitrogen. For the nine sub-basins, annual nitrogen depositions from both—all EMEP emission sources and only Polish emission sources—were calculated for each year of the period 1995–2014.

**Fig. 9:**
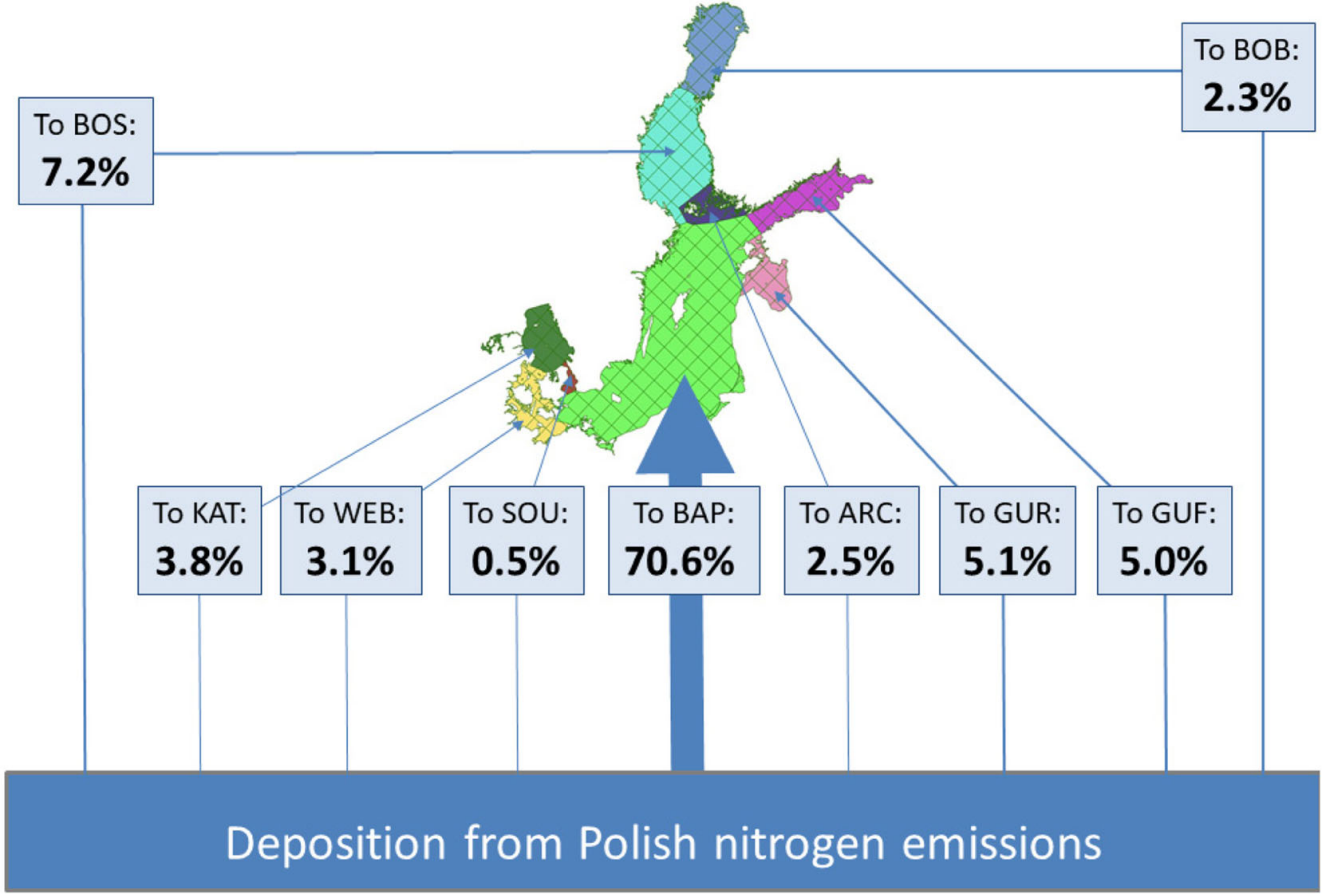
Distribution of nitrogen deposition from Polish emissions to nine sub-basins. The average values for the period 1995–2014 are presented

Nitrogen emitted from Polish sources is deposited over the entire EMEP domain and only a part of Polish emissions is deposited on the Baltic Sea basin and its sub-basins. This part varies from one year to another in the period 1995–2014, with minimum in the year 1997–5.0%, and maximum in 2012–8.3%. On average, for the entire period, 6.6% of Polish nitrogen emissions are deposited to the Baltic Sea basin. The relative distribution of nitrogen emitted from Polish sources over annual nitrogen deposition to nine selected sub-basins was also calculated for the period 1995–2014. The relative means percent of total deposition to the Baltic Sea basin from the Polish sources. The average, minimum, and maximum percent of Polish nitrogen emissions deposited in each sub-basin is shown in Fig. 9.

Baltic Proper is by far the largest sub-basin covering 50.1% of the entire Baltic Sea basin surface. Sub-basin Bothnian Sea is the next largest, covering 15.7% of the Baltic Sea Basin area, followed by Bothnian Bay (8.7%), Gulf of Finland (7.2%), and Kattegat (5.7%). The smallest sub-basin—The Sound—covers only 0.6% of the Baltic Sea area. Therefore, it is not a surprise that the sub-basin Baltic Proper is the main destination for deposition of nitrogen emitted in Poland with average annual contribution of 70.6% of the total annual Polish emission deposited to the Baltic Sea basin. On the other hand, The Sound sub-basin is the least probable destination of Polish nitrogen emissions with average contribution 0.5%. In general, more than 90% of nitrogen deposited into the Baltic Sea from Polish emissions is ending up in sub-basins located in the Central, North, and East Baltic Sea. Concerning variability of the Polish distribution, Baltic Proper sub-basin is the most stable with the range 67.3% in 2004 to 74.7% in 2007. The largest variability can be noticed for Western Baltic Sub-basin with the range 1.9% in 2012 to 5.8% in 2002.

**Fig. 10:**
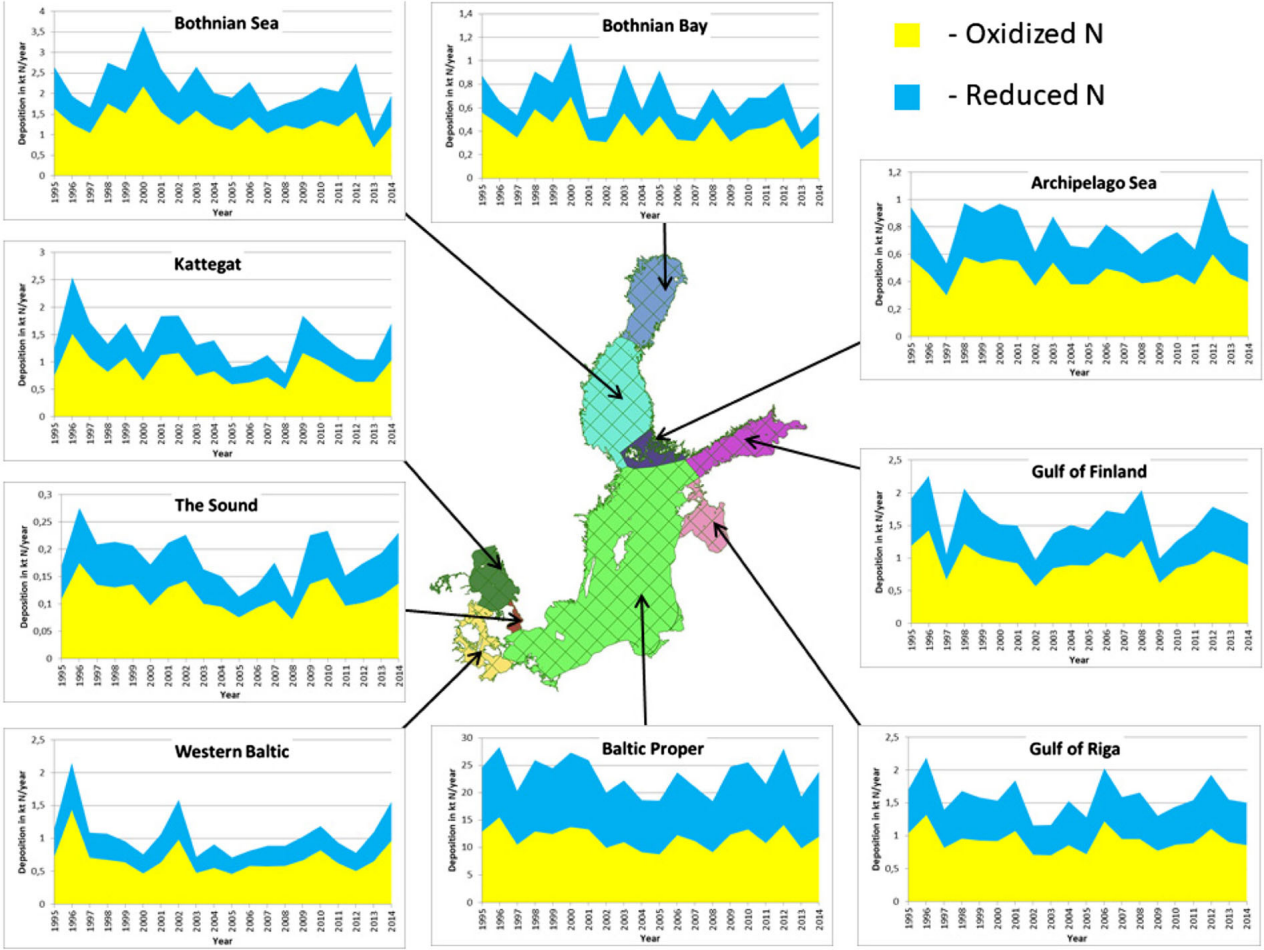
Time series of annual depositions of oxidized, reduced, and total nitrogen from Polish emission sources to individual sub-basins of the Baltic Sea in the period 1995–2014. Different deposition scales are used for different sub-basins

One of the important results of EMEP calculations requested every year by HELCOM is the information about long-term depositions of oxidized and reduced nitrogen to each of nine sub-basins of the Baltic Sea. The time series with depositions from Polish emission sources are presented in Fig. 10 for the period 1995–2014.

There are large differences in depositions to individual sub-basins and therefore different scales were used for different sub-basins. However, in all of them, deposition of oxidized nitrogen from Polish sources is higher than deposition of reduced nitrogen. Also, wet deposition into all sub-basins is higher than dry deposition of nitrogen emitted from Polish sources.

As expected, the largest deposition of total (oxidized + reduced) nitrogen from Polish sources can be noticed for the largest sub-basin, Baltic Proper, with the minimum value of 18 kt N in 2008 and maximum value 28 kt N in 1966. Minima of nitrogen deposition can be noticed for the smallest sub-basin (The Sound) ranging from 0.11 kt N in 2008 to 0.28 kt N in 1996.

There is a large variability in annual nitrogen deposition from Polish sources in the considered period. This variability is slightly larger in case of reduced nitrogen deposition than oxidized nitrogen deposition. It is mainly caused by changing meteorological conditions for atmospheric transport from one year to another.

No clear trend in nitrogen deposition from Polish sources can be observed for the considered period; however, for most of the sub-basins, annual deposition in 2014 is lower than annual deposition in the year 1995. In case of oxidized nitrogen, annual deposition is higher in 2014 in three sub-basins located in the west of the Baltic Sea: Kattegat (37%), Western Baltic (36%), and The Sound (51%). It is lower in all remaining sub-basins in the range 6–35%.

Annual deposition of reduced nitrogen emitted from Polish sources is significantly higher in 2014 than in 1995 in three sub-basins: The Sound (51%), Western Baltic (39%), and Kattegat (33%). Depositions to the Baltic Proper and Gulf of Riga remain on the same level, whereas depositions to four remaining sub-basins are lower (12–37%) in 2014 than in 1995.

Concerning annual depositions of total (oxidized + reduced) nitrogen, there is a clear increase in three sub-basins located in the west of the Baltic Sea: Western Baltic (37%), Kattegat (36%), and The Sound (35%). There is a decline of total nitrogen deposition between 1995 and 2014 in all remaining sub-basins with the most significant decline, 36%, in the Bothnian Sea sub-basin.

**Fig. 11:**
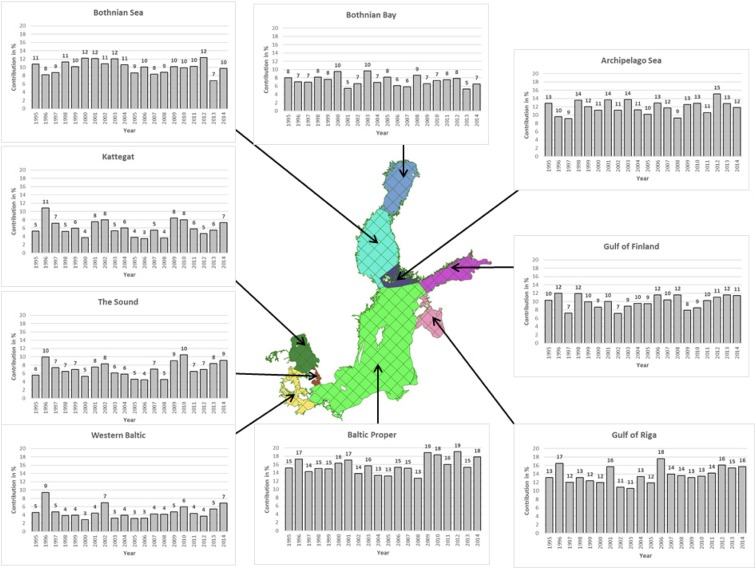
Polish contributions to annual total nitrogen depositions (sum of dry wet deposition of oxidized and reduced nitrogen) to the sub-basins of the Baltic Sea, in the period 1995–2014. Units: % of the total nitrogen deposition to each sub-basin from all EMEP emission sources

The most important from the decision-making point of view are Polish contributions to deposition of annual total nitrogen (sum of oxidized and reduced) to individual sub-basins of the Baltic Sea. These contributions for the period 1995–2014 are shown in Fig. 11 as percent of annual total nitrogen deposition to each sub-basin. For easier comparison, the same contribution scale is used for all sub-basins.

As expected a major contribution for Polish sources to total nitrogen deposition can be noticed for Baltic Proper sub-basin with the range 13–19%. In general, there is more contribution from Polish sources to the northern sub-basins: Gulf of Riga (11–18%), Archipelago Sea (9–15%), and Bothnian Sea (7–12%). Polish contributions to three western sub-basins are lower, with the lowest contribution (3–9%) to the sub-basin Western Baltic. This pattern is consistent with typical meteorological conditions in the present climate with prevailing westerly winds over the region. It is also consistent with the distribution of nitrogen emissions in the Baltic Sea region with German emissions dominating the deposition contributions to western sub-basins.

Polish contributions to annual depositions are changing from one year to another. Most stable are the contributions to the Baltic Proper sub-basin, relatively stable are contributions to north-east sub-basins, and least stable with largest inter-annual variability are Polish contributions to western sub-basins. Compared to the year 1995, Polish contributions to annual total nitrogen depositions in 2014 increased in six out of nine sub-basins. They were slightly (1%) lower in 2014 in three northern sub-basins: Archipelago Sea, Bothnian Sea, and Bothnian Bay.

## Contribution of Poland to Nitrogen Deposition in 2013

The results of the EMEP MSC-W model with 50-km resolution were compared with the results of the IMWM model with 14-km resolution. Comparison was performed for one year, 2013, for which the results of IMWM model were available.

### Results of the EMEP Standard Model

**Fig. 12:**
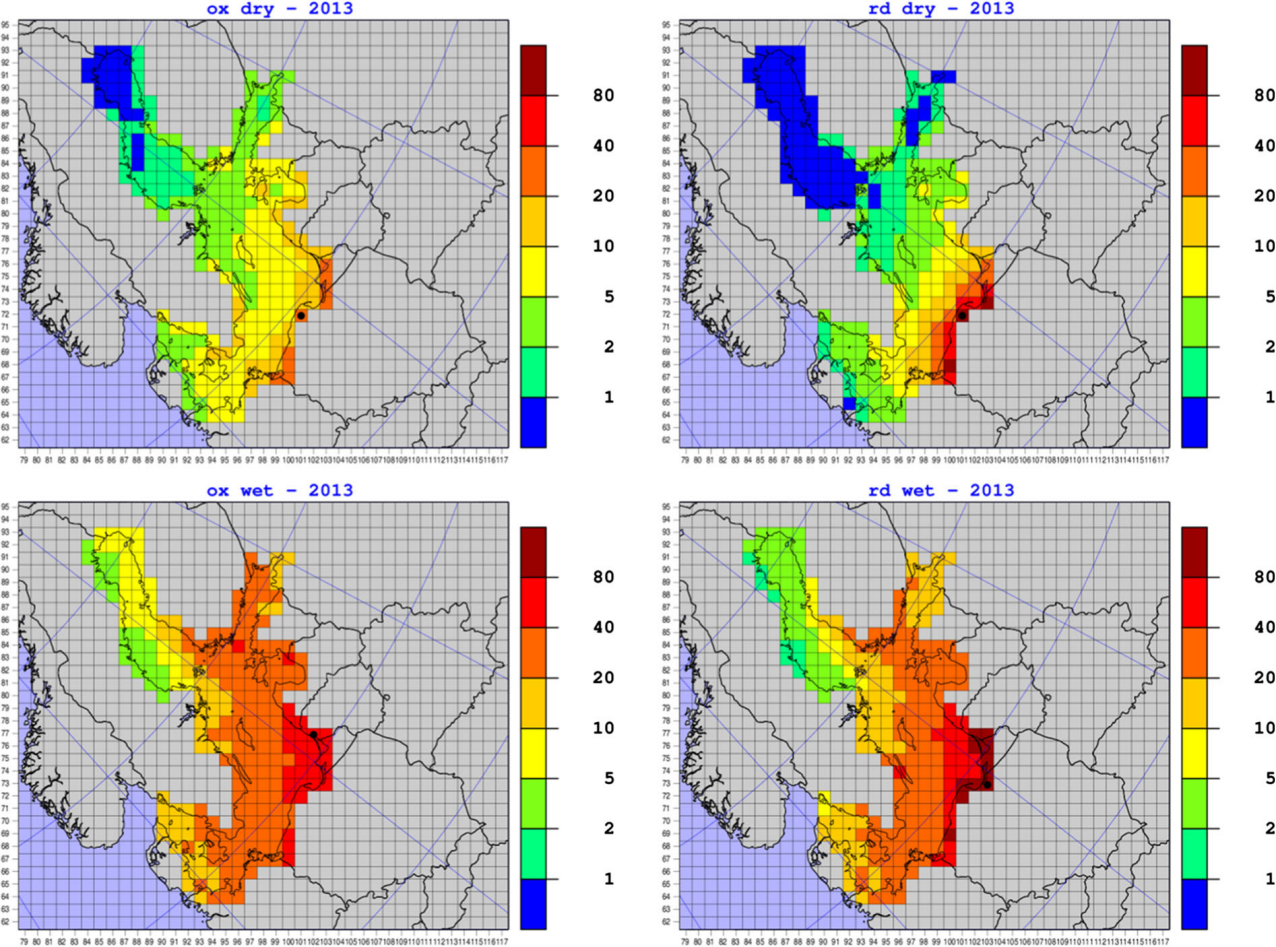
Maps with annual dry deposition of oxidized nitrogen (ox dry), dry deposition of reduced nitrogen (rd dry), wet deposition of oxidized nitrogen (ox wet), and wet deposition of reduced nitrogen (rd wet) in 2013. Results of the EMEP MSC-W model with 50-km resolution. Units: tons (N) per grid. A grid with maximum location is marked with a black circle

The results of the EMEP MSC-W standard model with 50-km resolution available for the year 2013 (Semeena et al. 2015) are presented in Fig. 12 as maps with annual dry deposition of oxidized nitrogen, dry deposition of reduced nitrogen, wet deposition of oxidized nitrogen, and wet deposition of reduced nitrogen into the Baltic Sea basin.

The maps in Fig. 12 confirm that wet deposition dominates over dry in the contributions from Polish emission sources, with wet deposition of oxidized nitrogen being the most effective. Polish contributions are less visible in dry deposition of nitrogen than in wet deposition. Dry deposition of reduced nitrogen is practically on the same level as dry deposition of oxidized nitrogen, but the gradient of the deposition is steeper in case of reduced nitrogen.

In all maps, there is a clear gradient of depositions from south to north of the Baltic Sea basin. However, this gradient is steeper in case of dry than wet deposition and steeper in case of reduced than oxidized nitrogen deposition. Maxima of most depositions are located on the coast of Poland, close to Polish emission sources. The only exception is the maximum of wet deposition of oxidized nitrogen which is located on the coast of Lithuania, also relatively close to the sources of Polish emissions. These maxima are marked with the black circles on the maps in Fig. 12. The values of maxima of oxidized dry, oxidized wet, reduced dry, and reduced wet annual depositions from Polish emission sources in 2013 are 37.1, 71.0, 134.5, and 155.1 mg m^−3^ for dry deposition of oxidized nitrogen, wet deposition of oxidized nitrogen, dry deposition of reduced nitrogen, and wet deposition of reduced nitrogen, respectively.

### Results of the IMWM Model

**Fig. 13:**
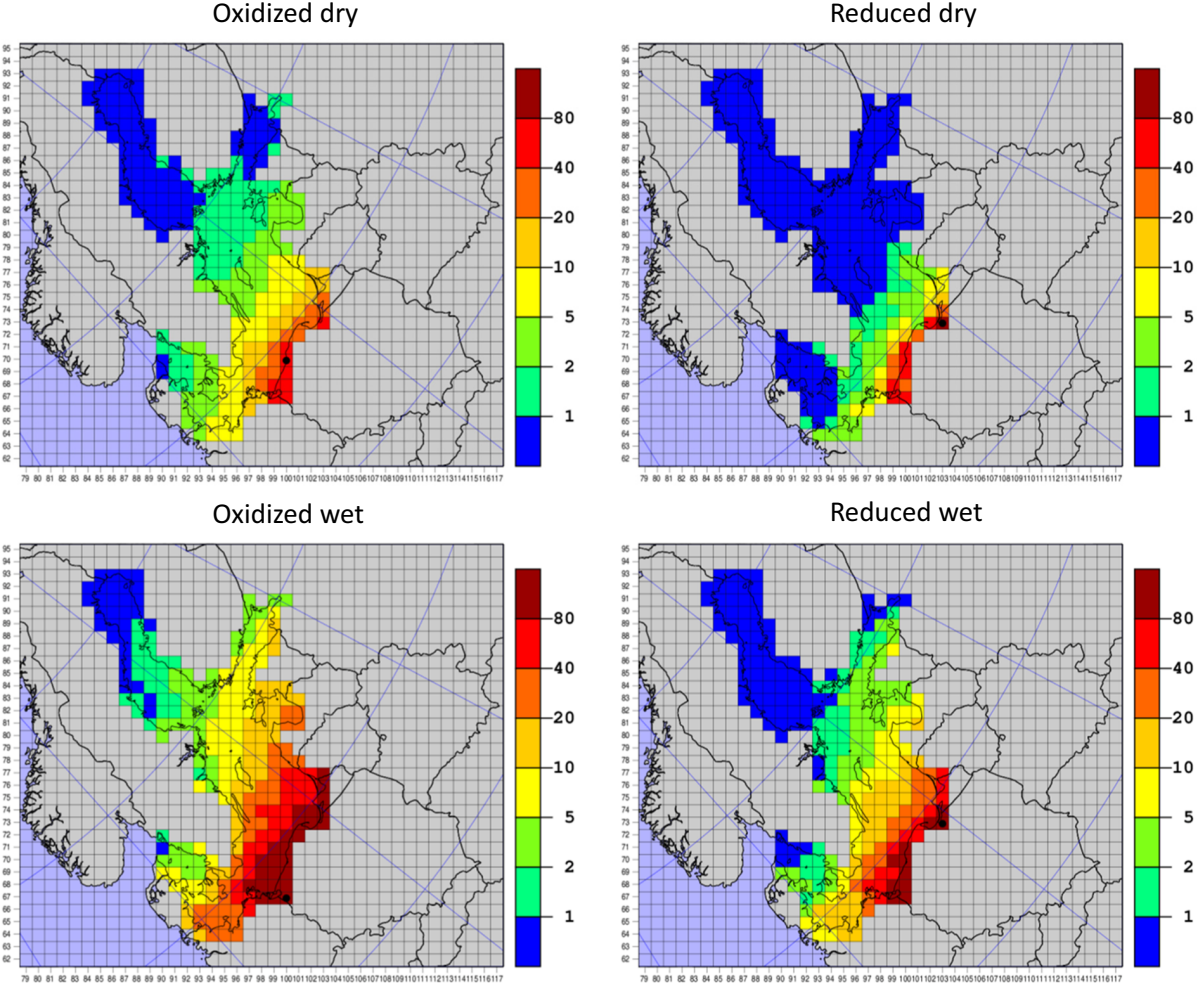
Maps with annual nitrogen depositions in 2013. Results of the IMWM model with 14-km resolution but presented in the standard EMEP grid with 50-km resolution for easier comparison. Units: tons (N) per grid. A grid with maximum location is marked with a black circle

Nitrogen depositions to the Baltic Sea in the year 2013 have been calculated with the IMWM model developed at the Institute of Meteorology and Water management in Warsaw, Poland (Mazur 2008; Mazur et al. 2014). Spatial distribution of annual dry deposition of oxidized nitrogen, wet deposition of oxidized nitrogen, dry deposition of reduced nitrogen, and wet deposition of reduced nitrogen from the IMWM model results for the year 2013 are shown in Fig. 13.

In case of the IMWM model, the contribution of Polish emission sources to annual nitrogen deposition in 2013 is mostly visible in wet deposition of oxidized nitrogen, followed by wet deposition of reduced nitrogen, dry deposition of oxidized nitrogen, and dry deposition of reduced nitrogen. In the results of the IMWM model, there is more Polish contribution in oxidized nitrogen deposition than in reduced nitrogen deposition. The maxima of all depositions are located on the coast of Poland, close to Polish emission sources.

As in case of EMEP model, in all maps in Fig. 13, a clear deposition gradient from south to north is present. This gradient is stronger in wet than dry deposition and approximately the same in depositions of oxidized and reduced nitrogen. The maxima of dry deposition of oxidized nitrogen, wet deposition of oxidized nitrogen, dry deposition of reduced nitrogen, and wet deposition of reduced nitrogen emitted from Polish emission sources in 2013 are 58.9, 271.1, 84.1, and 219.6 mg m^−3^, respectively.

### Comparison of the EMEP and IMWM Model Results

When comparing the results of EMEP and IMWM model some important differences between them, in addition to spatial resolution, should be taken into account. These two models use different sets of chemical reactions, different parameterizations of wet and dry deposition, different initial and boundary conditions, and different meteorological data as input. The emission of nitrogen data is the same in terms of country annual total values, but the spatial distribution of emissions is slightly different because of different numerical grids and spatial resolutions in both models. In addition, the way of computing Polish contributions to nitrogen deposition was different for two models. Having all these differences in mind, we do not expect a perfect agreement between EMEP and IMWM model results.

**Fig. 14:**
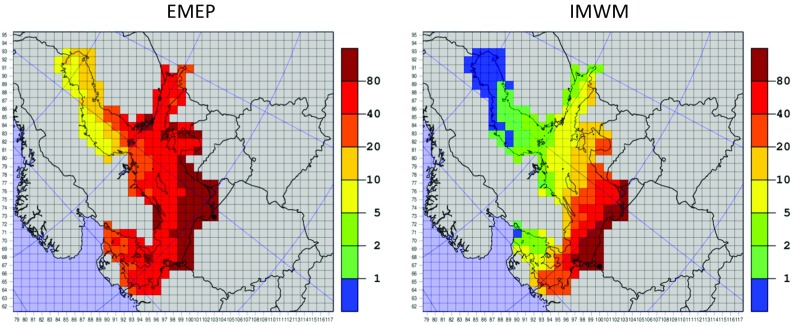
Maps with annual depositions of total nitrogen in 2013. Results of the EMEP MSC-W model with 50-km resolution and IMWM model with 14-km resolution both presented in the standard EMEP grid with 50-km resolution. Units: tons (N) per grid. A grid with maximum location is marked with a black circle

Some differences between EMEP and IMWM model are clearly visible in comparison of Figs. 12 and 13. The deposition gradient in the IMWM model results is higher than in the EMEP model results, indicating that the long-range atmospheric transport of nitrogen from Polish emission sources in EMEP MSC-W model is more effective than in case of the IMWM model. This fact is also confirmed by higher deposition maxima of the IMWM model, located close to maxima of the EMEP MSC-W model. In addition, it is confirmed in deposition maps of total nitrogen from both models shown in Fig. 14.

Comparison of annual 2013 nitrogen depositions to the Baltic Sea basin, as calculated from the EMEP and IMWM models, indicates that both models give quite similar results for oxidized nitrogen deposition, with slightly higher values from the IMWM model: 1% for dry deposition and 10% for wet deposition. In case of reduced nitrogen, the IMWM model gives much lower results: 56% for dry deposition and 49% for wet deposition. In comparison with the EMEP model, the IMWM model largely underestimates dry deposition (28%) and slightly less wet deposition (17%). Concerning annual total nitrogen deposition to the Baltic Sea basin in 2013, the IMWM model gives 25% lower value (20.3 kt N) than the EMEP MSC-W model (27.0 kt N).

## Discussion and Conclusions

There are two most important factors which determine nitrogen deposition from Polish sources: Polish nitrogen emissions and meteorological conditions during atmospheric transport from Poland. In addition, nitrogen emissions from other than Polish sources contributing to nitrogen deposition to the Baltic Sea are also important, especially for estimating the relative Polish contributions.

Agriculture, combustion, and transportation are the main sources of atmospheric nitrogen oxide and ammonia emissions in Poland (Fig. 3). Altogether, they contribute more than 97% to annual emissions of total (nitrogen oxides + ammonia) nitrogen from Poland in the period 1995–2014, with agriculture being the largest contributor (42–50%), followed by combustion (26–38%) and transportation (19–25%). Therefore, reduction of Polish contribution to nitrogen deposition is mainly dependent on the reduction of nitrogen emissions from those three categories. In case of combustion, SNAP sector 1 is the major source responsible for 63–70% of combustion emissions.

There is a visible reduction in Polish nitrogen oxides and ammonia emissions in the period 1995–2014 (Fig. 4). There are two periods, 2002–2007 for nitrogen oxides and 2004–2007 for ammonia, when Polish emissions are slightly increasing, but compared to the year 1995, nitrogen oxide and ammonia emissions in 2014 are 32 and 16% lower, respectively. The reduction of total nitrogen emissions from Poland between 1995 and 2014 is 25%.

Having these reductions of Polish emissions in mind, we expected similar reductions in annual nitrogen depositions to the Baltic Sea basin from Polish emission sources in the period 1995–2014. However, compared to emission, depositions from Polish emission sources to the entire Baltic Sea basin show much larger inter-annual variability in the analyzed period. In addition, there is only a small 5% decrease in total nitrogen deposition from the Polish sources between 1995 and 2014. The decrease of nitrogen deposition in the period 1995–2014 calculated by the EMEP MSC-W model is significantly smaller than nitrogen emission reductions in the same period. The main reasons for these differences in emission and deposition patterns are the differences in meteorological conditions for these two selected years and the inter-annual variability in meteorological conditions in general. For example, Polish annual emissions of total nitrogen decrease 5% between the year 2013 and 2014, but deposition from Polish sources increases 23% in the same period. If we calculated the change of deposition from Polish sources in the period 1995–2013 instead of 1995–2014, it will decrease 23% instead of 5%. This example illustrates how important meteorological conditions are. To reduce the effects of meteorology in calculated depositions, the so-called weather-normalized depositions are calculated in the joint EMEP-HELCOM project (Bartnicki et al. 2017). The temporal pattern of weather-normalized depositions follows much closer the emission pattern.

Comparison of time series of nitrogen depositions to the Baltic Sea basin from all EMEP emission sources and from only Polish sources (Fig. 6) shows similar temporal patterns and similar proportions between different kinds of nitrogen deposition. However, the reduction in depositions in oxidized nitrogen from all EMEP sources (27%) is slightly lower than the reduction of depositions from only Polish sources (32%) in the considered period, indicating possible decline of relative contributions from Polish sources to deposition of oxidized nitrogen. At the same time, there is no reduction in reduced nitrogen deposition from all EMEP sources with 1% increase between 1995 and 2014, also indicating possible decline of relative contributions from Polish sources.

This is not quite reflected in Fig. 7 presenting time series of relative Polish contributions to nitrogen deposition in the period 1995–2014, with a slight tendency of increasing at the end of the period. However, this increase is clearly lower than changes between different years caused by variable meteorological conditions. The range of Polish contribution to total nitrogen deposition in the considered period is from 10% in 2005 to 15% in 2012. The relative Polish contributions to nitrogen depositions in the period 1995–2014 are most effective in wet deposition of reduced nitrogen (13–18%), followed by wet deposition of oxidized nitrogen (9–15%), dry deposition of reduced nitrogen (8–14%), and oxidized dry deposition of oxidized nitrogen (6–13%).

Concerning sub-basins of the Baltic Sea, there are several factors responsible for the distribution of nitrogen deposition from Polish emission sources. The first one is the area of the sub-basin. Second is the distance from the Polish sources and the third is the direction and frequency of the atmospheric transport from Polish emission sources during a given year. In addition, the distribution of precipitation plays an important role in the deposition distribution since wet deposition is more effective than dry deposition of nitrogen. In the period 1995–2014, two sub-basins with the largest area—Baltic Proper and Gulf of Bothnia—received most of the nitrogen emitted from Poland. On the other hand, the lowest deposition from Polish sources can be noticed for The Sound sub-basin with the smallest area. On average, 71% of Polish annual nitrogen emission is deposited to the Baltic Proper sub-basin (Fig. 9) which covers only 50% of the entire Baltic Sea basin. This difference indicates an important role of transport distance and direction in the distribution of nitrogen deposition from Polish sources.

There is a large variability in annual depositions of oxidized and reduced nitrogen from Polish sources to individual sub-basins (Fig. 10). However, on average over the entire period, in all of them, deposition of oxidized nitrogen from Polish sources is higher than deposition of reduced nitrogen. Also, in all of them, wet deposition is higher than dry deposition of nitrogen emitted from Polish sources.

In absolute terms, the largest annual deposition of total nitrogen from Polish sources, on average 26 kt N, can be seen for the largest sub-basin (Baltic Proper) and the lowest annual deposition, on average 0.19 kt N, for the smallest sub-basin (The Sound).

In addition to meteorological conditions, a large variability in annual nitrogen deposition from Polish sources to individual sub-basins depends on several factors like size of the receptor distance from the source and direction of the transport. The lowest variability can be noticed for the largest sub-basin (Baltic Proper) and highest for the second largest sub-basin (Bothnian Sea), indicating that size of the receptor is not always the most important.

In eight out of nine sub-basins, deposition of oxidized nitrogen slightly dominates over deposition of reduced nitrogen, from 59% of total deposition in Gulf of Finland to 64% in Western Baltic. In only one sub-basin—Baltic Proper—depositions of oxidized and reduced nitrogen are on the same level with deposition of oxidized nitrogen accounting for 51% of total nitrogen deposition. Baltic Proper is a special sub-basin being the largest and the nearest to Polish emission sources of nitrogen and especially sources of reduced nitrogen. Relatively short transport distance is the main reason why Polish contribution to reduced nitrogen deposition is higher than in the remaining sub-basins.

An important indicator from the decision-makers’ point of view is annual total deposition of nitrogen to individual sub-basins as shown in Fig. 11. The amount of total nitrogen deposited to the sub-basins depends mainly on the amount of emission from the source region, distance from the source region, and local meteorological conditions. This implies that a larger source can potentially contribute more to the deposition even if it is located farther from the receptor region depending on the meteorological conditions of the particular day. In general, there is more variability in Polish contributions to western sub-basins than in contributions to remaining sub-basins. Compared to the year 1995, the relative contribution of Polish sources is slightly higher in the year 2014 in six out of nine sub-basins and it is slightly lower in three northern sub-basins: Archipelago Sea, Bothnian Sea, and Bay of Bothnia. Polish contributions in 2014 remain on the same level as in 1995 within the range (− 1, + 4%).

A clear decreasing south–north gradient in depositions of nitrogen calculated by EMEP MSC-W model is visible in the Baltic Sea basin which is more prominent in case of wet depositions as well as in the case of reduced nitrogen than oxidized nitrogen. As explained above, the maximum depositions are located closer to the Polish emission sources with an exception of maximum wet deposition of oxidized nitrogen over the coast of Lithuania.

A south-north gradient is also clearly present in annual 2013 nitrogen depositions calculated with the IMWM model with a better spatial resolution than the EMEP MSC-W model (Fig. 13). Here, the gradient is stronger in wet than dry deposition of nitrogen and approximately the same in oxidized and reduced nitrogen depositions. In the results of the IMWM model, maxima of all depositions are located on the coast of Poland. The stronger gradient in IMWM model indicates that atmospheric transport of nitrogen from Polish sources is longer in the results of EMEP MSC-W model than in the results of IMWM model.

Concerning annual 2013 input of nitrogen to the Baltic Sea basin, both models give quite similar results for deposition of oxidized nitrogen. But there are relatively larger differences in case of reduced nitrogen. The IMWM model gives significantly lower deposition of nitrogen to the Baltic Sea basin compared to that calculated by the EME MSC-W model: 56% for dry deposition of reduced nitrogen and 49% for wet deposition. In case of total nitrogen deposition to the Baltic Sea basin in 2013, the IMWM model gives 25% lower value (20.3 kt) than the EMEP MSC-W model (25.3 kt).

The differences between EMEP and IMWM model are not surprising since they use different parameterizations and different meteorological data. However, differences in deposition of reduced nitrogen are of concern with the IMWM model predicting approximately half of the annual reduced nitrogen deposition from Polish source to the Baltic Sea basin, compared to the EMEP MSC-W model. A question is which model is closer to the reality considering that IMWM model has much better spatial resolution (14 km) than the EMEP MSC-W model (50 km). We assume that the EMEP MSC-W model is more correct in this case for two reasons. First, comparison of two versions of the EMEP MSC-W model, standard with 50-km resolution and experimental (0.1° × 0.1° in geographical coordinates) with approximately 11-km resolution at 60°N, performed for the year 2013 (Simpson et al. 2015) showed good (within 10%) agreement between calculated nitrogen depositions. The second reason is a continuous evaluation of the EMEP model by comparing its results with measurements in over 100 European stations including so-called HELCOM station in the Baltic Sea region (Gauss et al. 2015). The IMWM model has also been verified against measurements (Mazur 2008, 2016), but not so much in the Baltic Sea region. Both EMEP and IMWM models use the same nitrogen emission data for the year 2013, however in different spatial resolution. The resolution can create some differences in the depositions, but judging from the experiments with spatial resolution of the EMEP model (Gauss et al. 2015), they are not significant. Detailed analysis of differences between the models is outside the scope of this study, but the most probable reasons for differences between the models are different parameterizations of chemical reactions and deposition processes, different methods for calculation of Polish contribution to nitrogen deposition, and different meteorological data used by both models. Since the differences are mostly visible in deposition of reduced nitrogen, different chemistry and different parameterizations of deposition processes are most likely the main reasons.

Polish emission sources have been and probably will remain one of the main contributors to atmospheric nitrogen deposition to the Baltic Sea among all EMEP emission sources. Therefore, it is important for the HELCOM activities to calculate as accurate as possible annual contribution of Polish sources to nitrogen deposition, both to Baltic Sea basin and its sub-basins.
